# Early experience with targeted and combination biopsies in prostate cancer work-up in Denmark from 2012 to 2016

**DOI:** 10.1007/s00345-024-05234-4

**Published:** 2024-09-14

**Authors:** Anna Arendt Blak, Hein V. Stroomberg, Klaus Brasso, Signe Benzon Larsen, Andreas Røder

**Affiliations:** 1 Copenhagen Prostate Cancer Center, Department of Urology, Copenhagen University Hospital - Rigshospitalet, Ole Maaløes Vej 24, Copenhagen, 7521. 2200 Denmark; 2 Section of Biostatistics, Department of Public Health, University of Copenhagen, Copenhagen, Denmark; 3 Section of Epidemiology, Department of Public Health, University of Copenhagen, Copenhagen, Denmark; 4 Department of Clinical Medicine, University of Copenhagen, Copenhagen, Denmark

**Keywords:** Prostate cancer, DaPCaR, MRI, Grading, Disease-specific mortality

## Abstract

**Purpose:**

To investigate the early implementation of combined systematic and targeted (cBx) primary biopsy in prostate cancer diagnosis and define the concordance in Gleason grading (GG) of different biopsy techniques with radical prostatectomy (RP) pathology.

**Methods:**

This population-based analysis includes data on all men in Denmark who underwent primary cBx or standalone systematic (sBx) prostate biopsy between 2012 and 2016. Biopsy results were compared to RP pathology if performed within a year. Concordance measurement was estimated using Cohen’s Kappa, and the cumulative incidence of cancer-specific death was estimated at 6 years with the Aalen-Johansen estimator.

**Results:**

Concordance between biopsy and RP pathology was 0.53 (95CI: 0.43–0.63), 0.38 (95CI: 0.29–0.48), and 0.16 (95CI: 0.11–0.21) for cBx, targeted biopsy (tBx), and sBx, respectively. For standalone sBx and RP, concordance was 0.29 (95CI: 0.27–0.32). Interrelated GG concordance between tBx and sBx was 0.67 (95CI: 0.62–0.71) in cBx. The proportion of correctly assessed GG based on RP pathology was 54% in both cBx and standalone sBx. Incidence of prostate cancer-specific death was 0% regardless of biopsy technique in GG 1, and 22% (95CI: 11–32), 30% (95CI: 15–44), and 19% (95CI: 7.0–30) in GG 5 for cBx, tBx, or sBx, respectively.

**Conclusion:**

Overall, the cBx strategy demonstrates higher concordance to RP pathology than the standalone sBx. However, cBx exhibits more overgrading of the GG of RP pathology compared to sBx. Ultimately, the classic grading system does not take change in the diagnostic pathway into account, and grading should be altered accordingly to ensure appropriate treatment.

**Supplementary Information:**

The online version contains supplementary material available at 10.1007/s00345-024-05234-4.

## Introduction

The implementation of magnetic resonance imaging (MRI) is perceived as the biggest advancement in prostate cancer diagnosis since systematic transrectal ultrasound (TRUS)-guided biopsies (sBx) were introduced [1]. The use of MRI enables clinicians to exclude men from unnecessary biopsies and allows for targeted biopsies (tBx), compared to previous systematic sampling of the entire prostate. While tBx is believed to be superior in detecting clinically significant cancer, it has also been discussed that tBx may lead to overgrading and subsequent overtreatment [2–5]. It has been reported that combining targeted and systematic biopsies (cBx) provides a higher detection rate of clinically significant prostate cancer than either biopsy strategy independently [6, 7]. Until now, the impact of the implementation of tBx and cBx on a nationwide level is unknown.

In this study, we evaluate the implementation of cBx in a population-based nationwide setting, including all Danish men who underwent their first prostate biopsy in the period 2012–2016. We aim to elucidate the accuracy of the different biopsy strategies by comparing them to radical prostatectomy (RP) histopathology. We further aim to gain insight into how the new approach compares to the previous standard standalone sBx.

## Materials and methods

**Fig. 1:**
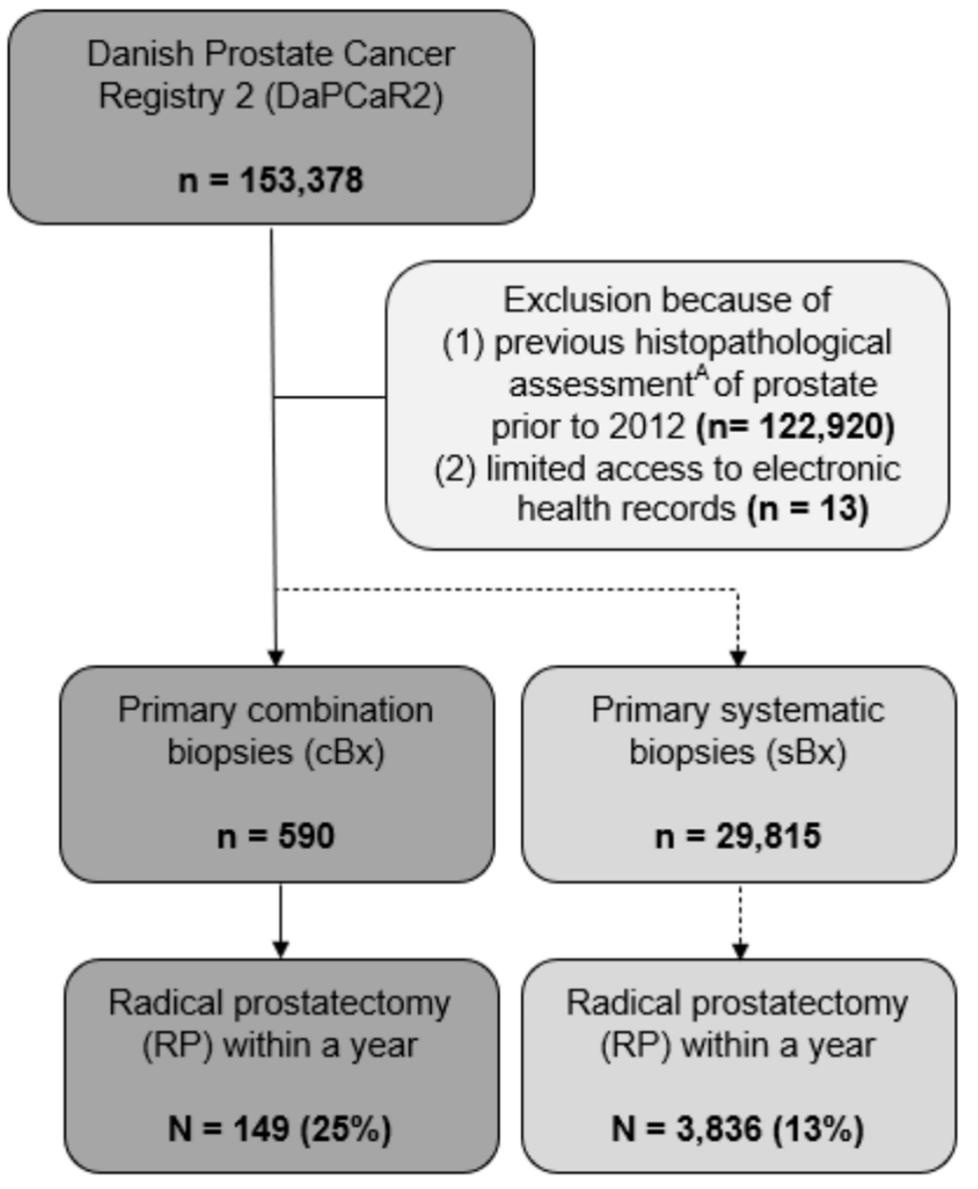
Flow chart of subject inclusion and exclusion. A = Any histopathological assessment of the prostate e.g. previous biopsies or transurethral resection of the prostate (TUR-P)

Data was extracted from the Danish Prostate Cancer Registry 2 (DaPCaR2) described in detail previously [8]. Shortly, the registry includes all histopathological assessments of prostate tissue in Denmark from 1995 to 2016. In this study, we examined all men with a primary cBx performed between January 1, 2012, and December 31, 2016 (Fig. 1). Men who migrated were excluded from the study. For men biopsied using cBx, patient characteristics, including prostate-specific antigen (PSA), and clinical stage were obtained by chart review. Characteristics of men who underwent standalone sBx were extracted directly from the DaPCaR database. From the national patient registry, we extracted all abdominal MRIs performed within 15 days of the standalone sBx [9]. To assess cBx, we extracted the Gleason grading (GG) of the tBx and sBx separately and recorded the highest GG as the cBx conclusion.

To evaluate the accuracy of cBx, we compared the biopsy GG with the final pathology following RP. The assessment was made by comparing GG on an ordinal scale (non-malignant, GG 1, GG 2, GG 3, GG 4, GG 5). Furthermore, concordance in GG from biopsy to RP specimen was assessed by up- and downgrading from biopsy to RP GG assessment. In cases where the final histopathological assessment was non-malignant, a distinction was made between downgrading to a lower GG and a non-malignant result.

In addition to the evaluation of biopsy accuracy, we included mortality rates according to GG of the tBx and sBx component of cBx, the cBx GG conclusion, and for GG defined by standalone sBx with localized disease.

### Statistical analyses

Descriptive characteristics were reported as the number of men, accompanied by the percentage for categorical variables and by median and interquartile range for continuous variables. Concordance was assessed by weighted Cohen’s kappa with 95% confidence intervals (95CI) [10]. A concordance of 0-0.20, 0.21–0.39, 0.40–0.59, 0.60–0.79, 0.80–0.90, and > 0.90 are considered none, minimal, weak, moderate, strong, and almost perfect, respectively. Concordance is assessed for GG of tBx and sBx that make up the cBx, and the GG of cBx, tBx, and sBx to subsequent RP pathology performed within a year of primary biopsy. As control the concordance of standalone sBx with RP when performed within a year is assessed. In the standalone sBx cohort, biopsy conclusions with unidentified adenocarcinoma, undifferentiated GG 2/3, and undifferentiated GG 4/5 were excluded.

The cumulative incidence of cause-specific death was estimated with the Aalen-Johansen estimator, where prostate cancer-specific death and other cause death were competing events. Men were followed until death, migration, or end of follow-up (February 9th, 2023), whichever came first. Median follow-up time is defined by median time to censoring. All analyses were performed using RStudio Version 1.4.1106 with R version 4.2.2.

## Results

### Patient characteristics

A total of 590 men had cBx and 29,815 had standalone sBx in Denmark from 2012 to 2016. Clinical characteristics are summarized in Table 1. Among men with primary cBx, 67% were diagnosed with prostate cancer, while the diagnostic rate was 67% for men with primary standalone sBx. A total of 149 men with cBx and 3,836 men with standalone sBx were treated with RP within a year of primary biopsy. Out of the men biopsied with standalone sBx, 345 (1.2%) had a recorded abdominal MRI within 15 days of biopsy without targeted biopsy.

**Table 1:** Clinical characteristics for men with a primary combination or systematic prostate biopsy in Denmark during 2012–2016

	Combination biopsy (cBx) *n* = 590	Standalone systematic biopsies (sBx) *n* = 29,815
Age, yr (median, IQR)	67 (62–71)	68 (63–73)
PSA, ng/ml (median, IQR)	7.8 (5.6–23)	8.5 (5.8–18)
MRI performed prior to biopsy, n (%)	590 (100%)	345 (1.2%)
**Number of targeted cores**		
One	302 (51%)	NA
Two	218 (37%)	NA
Three	61 (10%)	NA
Four	9 (1.5%)	NA
**Clinical T-category** ^**A**^		
T1a-T2a	382 (65%)	6,889 (23%)
T2b	52 (8.8%)	908 (3.0%)
T2c or higher	99 (17%)	8,368 (28%)
T2 unspecified	-	731 (2.5%)
Tx	57 (10%)	12,919 (43%)
**Biopsy conclusion** ^**B**^		
Non-malignant	189 (32%)	12,775 (43%)
GG 1	104 (18%)	4,067 (14%)
GG 2	155 (26%)	4,877 (16%)
GG 3	57 (10%)	2,261 (7.6%)
GG 4	33 (5.6%)	2,093 (7.0%)
GG 5	52 (8.8%)	3,658 (12%) ^C^
Neuroendocrine/small cell carcinoma	-	16 (< 0.1%)
**RP conclusion** ^**D**^	*n* = 149	*n* = 3,836
Non-malignant	< 5	14 (< 0.1%)
GG 1	< 5	432 (11%)
GG 2	62 (42%)	2,214 (58%)
GG 3	43 (29%)	801 (21%)
GG 4	12 (8.1%)	180 (4.7%)
GG 5	31 (21%)	195 (0.5%)

A: Clinical T-scores were only reported at the time of diagnosis, thus are not available in men with non-malignant, standalone sBx.

B: In cBx, the highest biopsy conclusion of either targeted or systematic biopsy was reported

C: Including biopsy conclusion of GG 4/5 (*n* = 573)

D: Conclusion of all RPs performed within a year of primary biopsy

PSA = prostate-specific antigen; T-category = tumor-category; GG = Gleason grading; RP = radical prostatectomy

### Concordance

The concordance between the tBx and sBx conclusions in cBx (*n* = 590) was 0.67 (95CI: 0.62–0.71), indicating moderate agreement between the two strategies. For all men with cBx and RP within a year the concordance with RP pathology was 0.53 (95CI: 0.43–0.63), 0.38 (95CI: 0.29–0.48), and 0.16 (95CI: 0.11–0.21), for cBx, tBx and sBx GG, respectively. This represents a weak concordance for cBx, a minimal concordance for tBx, and a no concordance for sBx. In our control cohort of men with standalone sBx and RP concordance was 0.29 (95CI: 0.27–0.32), representing minimal concordance (Supplementary Table 1).

### Grading

The assessment of up- and downgrading of cBx conclusion with subsequent RP pathology showed unchanged GG from cBx to RP in 54% of cases (Table 2). The unchanged GG of sBx and tBx of cBx was 49% and 45%. The control standalone sBx showed that 54% of cases were unchanged. For men diagnosed using cBx, downgrading was found to be 21%, 13%, and 13% and upgrading was found to be 25%, 38%, and 42% for overall cBx, sBx and tBx, respectively. For the control standalone sBx cohort downgrading was 16% and upgrading was 33%.

### Mortality

**Fig. 2:**
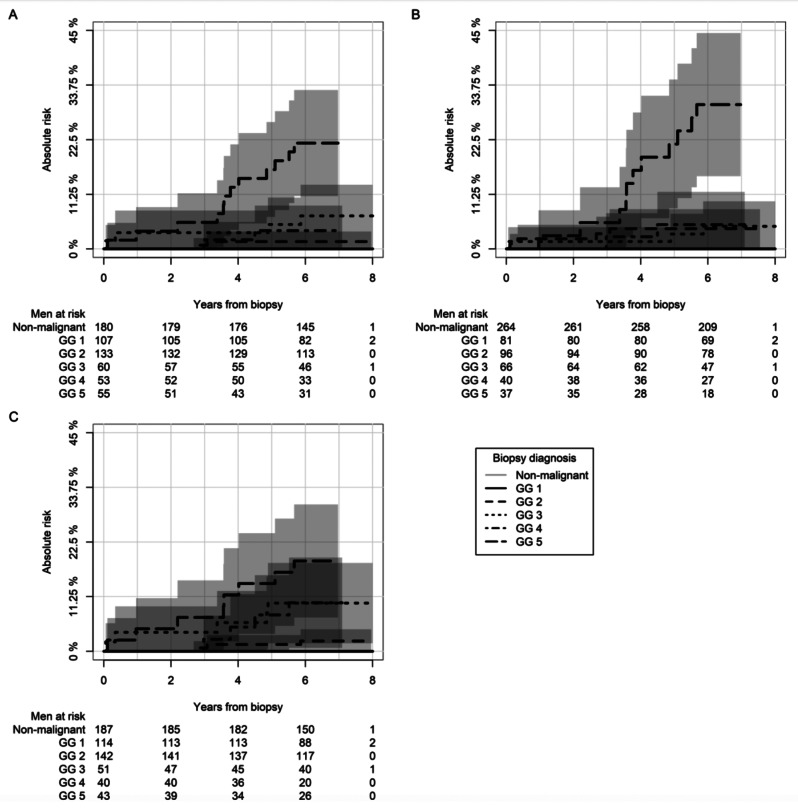
Absolute risk of prostate cancer-specific death since the time of primary combined biopsy (cBx) stratified biopsy Gleason grading (GG) in overall highest GG (A), targeted component (B), and systematic component (C)

The median follow-up time was 6.2 years (IQR: 6.0-6.7) for men with a defined GG in cBx (*n* = 590). In all three biopsy strategies the risk of prostate cancer-specific death at the 6-year mark was 0% in men with non-malignant or GG 1 conclusion (Fig. 2). Among men diagnosed with GG 2 in cBx, tBx, and sBx the prostate cancer-specific death was 1.5% (95CI: 0-3.6), 4.2% (95CI: 1.7–8.2), and 2.1% (95CI: 0-4.5), respectively. Among men diagnosed with GG 3 in cBx, tBx, and sBx the prostate cancer-specific death was 6.8% (95CI: 0.4–13), 4.7% (95CI: 0-9.8), and 9.9% (95CI: 1.7–18), respectively. Among men diagnosed with GG 4 in cBx, tBx, and sBx the prostate cancer-specific death was 3.8% (95CI: 0-8.9), 5.0% (95CI: 0–12), and 10% (95CI: 0.7–19), respectively. Among men diagnosed with GG 5 in cBx, tBx, and sBx the prostate cancer-specific death was 22% (95CI: 11–33), 30% (95CI: 15–44), and 19% (95CI: 7.0–30), respectively.

For men with standalone sBx, the 6-year risk of prostate cancer-specific death was 1.4% (95CI: 1.0-1.7), 3.7% (95CI: 3.2–4.2), 12% (95CI: 10–13), 18% (95CI: 16–20), and 33% (95CI: 32–35), when diagnosed with GG1, GG2, GG3, GG4, or GG5, respectively (Supplementary Fig. 1).

## Discussion

In this nationwide population-based study on early adoption of MRI in the diagnostic pathway, we showed that cBx demonstrated the highest concordance from biopsy to RP pathology, whereas both tBx and sBx demonstrated below weak concordance. Additionally, the interrelated concordance between tBx and sBx in cBx is moderate. Cancer-specific death was as expected low at the 6-year follow-up for most diagnostic GG. However, as expected men diagnosed with GG 5, faced a high 6-year risk of cancer-specific death, this being regardless of biopsy strategy.

In Denmark, MRI pre-biopsy has been gradually introduced and is now considered as the standard of care in diagnostic work-up by providing information on which men are eligible for tBx. The initial purpose of including MRI in the diagnostic pathway was to decrease the total number of men directed for biopsy and to reduce the number of biopsies performed per subject subsequently decreasing diagnosis of low-risk prostate cancer. The United Kingdom has largely adopted the use of pre-biopsy MRI selection and subsequent standalone tBx, resulting in a lowering in number of men being diagnosed with GG 1 in recent years [11]. However, this approach is still debated, as the long-term prognostic value of a negative MRI is largely unknown in contrast to the prognostic value of non-malignant biopsies [12]. In general, our results support a diagnostic pathway using MRI along with either a tBx- or -cBx pathway, as the overall performance of these two strategies seems superior to sBx based on the parameters measured. Recent MRI screening trials among men with a PSA above 3 ng/ml demonstrate a high concordance between positive MRI findings and tBx with clinically significant cancer further supporting this claim [13, 14]. Although cBx exhibits the highest concordance, this method also requires the highest number of biopsies, and thus the men might experience more discomfort. However, according to a recent study, there is no significant increase in the risk of post-biopsy infection when comparing sBx to cBx [15]. Ultimately, the adoption of transperineal biopsies will likely nearly eliminate the risk of infections, but more data is needed on grade migration due to the difference between the transperineal and transrectal biopsy techniques [16].

Based on our data, cBx is the optimal biopsy strategy for predicting the “true” GG in a subsequent RP pathology. However, we find that this strategy also results in the highest amount of overgrading. Importantly, the prognostic value of the biopsy conclusion decreases when men are biopsied with strategies that lead to higher GG. This is a classic example of the Will-Rogers effect, and it needs to be questioned whether this is desirable [17]. It can be argued that these differences are due to differences in cancer load for the same GG in the different biopsy strategies. It is current practice to take the highest GG of either biopsy strategy to use as the final GG conclusion of cBx [11]. When looking at our data, it should be speculated that the optimal GG grading is not the highest but rather an intermediate GG between the two strategies. This combined with the ISUP 2019 system in Denmark would effectively reduce the risk of overgrading [18]. It has previously been shown that over time improvements in biopsy technique, strategy, and grading increase concordance. Thus, this strategy should be tested on an individual patient level to assure higher concordance with the RP specimen to obtain the “true” GG grade at the time of diagnosis [19–21]. Moreover, the number of targeted cores taken per lesion is low and likely represents the early implementation period. In current practice, 3 to 4 biopsies are usually taken per lesion, which may increase the concordance of the tBx and cBx [22].

The strength of this study lies in the accessibility and veracity of the nationwide data due to the strong Danish registries and electronic health registrations. This study is the first to evaluate the nationwide use of tBx and provides a unique insight into the early implementation of this diagnostic pathway. As our data was collected from the same period that MRI and tBx were first introduced, it presents some limitations, most importantly the fact that the approach was still new, and consequently, few men underwent standalone tBx in the period, and PIRAD scoring was insufficient to report. As of now, the MRI-tBx diagnostic pathway is the standard practice in some medical centers in Denmark. Moreover, early implementation results included a clinical trial during the study period that examined a specific population of biopsy-naïve men with a suspicion of prostate cancer with MRI and performed subsequent tBx regardless of the grading of MRI lesions to target [23]. Arguably, these biopsies may not qualify as true tBx and thus we see an inflation of the concordance between tBx and sBx in cBx. This likely also explains the lower GG of tBx than expected [24]. This means that it is likely that more modern data would favor tBx but is unlikely to alter our conclusion. The comparison of the standalone sBx to the cBx is limited by the fact that cBx contained manual chart review and the standalone biopsies did not. However, considering the similarity of the results we do not believe this to have had a major influence. A small proportion of men were not directed to tBx after MRI, but proceeded to get sBx within 15 days of the scan. Unfortunately, detailed reports on the reasoning behind this are not available and physicians’ biases might also affect the decision-making.

Caution is warranted in interpreting the survival analyses as the sample size is small for the individual GG groups resulting in large uncertainty of the estimate. E.g. we find that mortality rates for men with GG 3 and 4 in sBx are twice that of cBx and tBx, however the confidence intervals are overlapping. Thus, this does not exclude the possibility that the mortality rates are the same. Nonetheless, the differences are large in general, and the effects are consistent in our results, indicating a true effect. Mortality of men biopsied with standalone sBx is largely comparable for most GG except for GG5, where we observe a higher risk of disease-specific death. This suggests that the use of MRI was initially implemented for men in lower-risk groups in Denmark.

## Conclusion

Conclusively, this unique nationwide cohort of early implementation of MRI suggests that the accuracy of biopsy regardless of methodology remains suboptimal with no to weak concordance. Moreover, we show a notable underestimation of GG from sBx to RP pathology, but alternatively some overestimation in cBx to RP pathology. Generally, cBx performed best overall, as men diagnosed with this strategy also exhibited the least cancer-specific mortality after 6 years of follow-up among men with GG 3 or above. To hopefully reduce this risk of overdiagnosis and -treatment, cBx grading should be optimized to ensure that the diagnostic grade group reflects that of the GG.

## Electronic supplementary material

Below is the link to the electronic supplementary material.

Supplementary Material 1

## Statements and Declarations

All authors contributed to the study conception and design. Material preparation, data collection and analysis were performed by Anna Arendt Blak, Hein Stroomberg and Andreas Røder. The first draft of the manuscript was written by Anna Arendt Blak, and all authors commented on previous versions of the manuscript. All authors read and approved the final manuscript. The authors did not receive support from any organization for the submitted work.
