# Supplementary material for: Early experience with targeted and combination biopsies in prostate cancer work-up in Denmark from 2012 to 2016
Source: World J Urol. 2024 Sep 14;42(1):523. doi: 10.1007/s00345-024-05234-4 (PMC11401785; doi:10.1007/s00345-024-05234-4)
Supplement: Supplementary file 1 — Supplementary Material 1 [file 345_2024_5234_MOESM1_ESM.docx]

**Supplementary Table 1:** Concordance between Gleason grading (GG) in men with a primary prostate biopsy and radical prostatectomy (RP) if performed within a year in Denmark during 2012-2016.

1A: From overall highest GG in cBx to RP. 1B: From sBx of cBx to RP. 1C: From tBx of cBx to RP. 1D: Interrelated concordance of histology result of tBx and sBx of cBx. 1E: From control standalone sBx to RP.

| **1A** | | | | | | | |
| --- | --- | --- | --- | --- | --- | --- | --- |
| **cBx**  **RP** | **Non-malignant** | **GG 1** | **GG 2** | **GG 3** | **GG 4** | **GG 5** | **Total** |
| **Non-malignant** | <5 | <5 | <5 | <5 | <5 | <5 | <5 |
| **GG 1** | <5 | <5 | <5 | <5 | <5 | <5 | <5 |
| **GG 2** | <5 | 8 | 38 | 12 | <5 | <5 | 62 |
| **GG 3** | <5 | <5 | 10 | 19 | 11 | <5 | 43 |
| **GG 4** | <5 | <5 | <5 | <5 | 5 | <5 | 12 |
| **GG 5** | <5 | <5 | <5 | 5 | 7 | 18 | 31 |
| **Total** | 1 | 10 | 53 | 36 | 25 | 24 | **149** |
| **1B** | | | | | | | |
| **sBx**  **RP** | **Non-malignant** | **GG 1** | **GG 2** | **GG 3** | **GG 4** | **GG 5** | **Total** |
| **Non-malignant** | <5 | <5 | 5 | <5 | <5 | <5 | <5 |
| **GG 1** | <5 | <5 | <5 | <5 | <5 | <5 | <5 |
| **GG 2** | <5 | 12 | 40 | 8 | <5 | <5 | 62 |
| **GG 3** | <5 | <5 | 13 | 15 | 8 | <5 | 43 |
| **GG 4** | <5 | <5 | 6 | <5 | <5 | <5 | 12 |
| **GG 5** | <5 | <5 | <5 | 6 | 7 | 14 | 31 |
| **Total** | 6 | 17 | 62 | 29 | 19 | 16 | **149** |
| **1C** | | | | | | | |
| **tBx**  **RP** | **Non-malignant** | **GG 1** | **GG 2** | **GG 3** | **GG 4** | **GG 5** | **Total** |
| **Non-malignant** | <5 | <5 | <5 | <5 | <5 | <5 | <5 |
| **GG 1** | <5 | <5 | <5 | <5 | <5 | <5 | <5 |
| **GG 2** | 5 | 15 | 32 | 7 | <5 | <5 | 62 |
| **GG 3** | 6 | <5 | 10 | 17 | 7 | <5 | 43 |
| **GG 4** | <5 | <5 | 5 | <5 | <5 | <5 | 12 |
| **GG 5** | <5 | <5 | <5 | 8 | 6 | 13 | 31 |
| **Total** | 14 | 18 | 48 | 33 | 18 | 18 | **149** |
| **1D** | | | | | | | |
| **sBx**  **tBx** | **Non-malignant** | **GG 1** | **GG 2** | **GG 3** | **GG 4** | **GG 5** | **Total** |
| **Non-malignant** | 172 | 52 | 22 | <5 | 5 | <5 | 259 |
| **GG 1** | 6 | 44 | 27 | <5 | <5 | <5 | 81 |
| **GG 2** | <5 | 13 | 67 | 9 | <5 | <5 | 99 |
| **GG 3** | <5 | <5 | 17 | 25 | 9 | 12 | 66 |
| **GG 4** | <5 | <5 | 6 | 9 | 18 | <5 | 41 |
| **GG 5** | <5 | <5 | <5 | <5 | 6 | 25 | 44 |
| **Total** | 192 | 115 | 142 | 53 | 41 | 47 | **590** |
| **1E** | | | | | | | |
| **sBx**  **RP** | **Non-malignant** | **GG 1** | **GG 2** | **GG 3** | **GG 4** | **GG 5** | **Total** |
| **Non-malignant** | <5 | <5 | 5 | <5 | <5 | <5 | 14 |
| **GG 1** | 46 | 270 | 105 | 8 | <5 | <5 | 432 |
| **GG 2** | 113 | 474 | 1,361 | 171 | 81 | 14 | 2,214 |
| **GG 3** | 29 | 63 | 304 | 295 | 80 | 30 | 801 |
| **GG 4** | 9 | 13 | 41 | 37 | 55 | 25 | 180 |
| **GG 5** | 7 | 9 | 21 | 43 | 48 | 67 | 195 |
| **Total** | 208 | 833 | 1,837 | 555 | 267 | 136 | **3,836** |

**Supplementary Table 2**: Up- and downgrading of Gleason grading (GG) from primary prostate biopsy after radical prostatectomy (RP) if performed within a year in men in Denmark during 2012-2016.

2A: From overall highest GG in combined biopsy (cBx) to RP. 2B: From sBx of cBx to RP. 2C: From tBx in cBx to RP. 2D: from control standalone sBx to RP.

| **2A** | | | | | | | |
| --- | --- | --- | --- | --- | --- | --- | --- |
| **cBx**  **RP** | **Non-malignant**  n = <5 | **GG 1**  n = 10 | **GG 2**  n = 53 | **GG 3**  n = 36 | **GG 4**  n = 25 | **GG 5**  n = 24 | **Total**  n = 149 |
| **Downgrading** | N/A | <5 | 0 | 12  (33%) | 13  (52%) | 6  (25%) | 31  (21%) |
| **Unchanged** | <5 | <5 | 38  (72%) | 19  (53%) | 5  (20%) | 18  (75%) | 81  (54%) |
| **Upgrading** | <5 | 9  (90%) | 15  (28%) | 5  (14%) | 7  (28%) | N/A | 37  (25%) |
| **2B** | | | | | | | |
| **sBx**  **RP** | **Non-malignant**  n = 6  (tBx pos. for cancer) | **GG 1**  n = 17 | **GG 2**  n = 62 | **GG 3**  n = 29 | **GG 4**  n = 19 | **GG 5**  n = 16 | **Total**  n = 149 |
| **Downgrading** | N/A | <5 | <5 | 8  (28%) | 9  (47%) | <5 | 19  (13%) |
| **Unchanged** | <5 | <5 | 40  (65%) | 15  (52%) | <5 | 12  (88%) | 73  (49%) |
| **Upgrading** | 6  (100%) | 16  (94%) | 22  (35%) | 6  (21%) | 7  (37%) | N/A | 58  (38%) |
| **2C** | | | | | | | |
| **tBx**  **RP** | **Non-malignant**  n = 14  (sBx pos. for cancer) | **GG 1**  n = 18 | **GG 2**  n = 48 | **GG 3**  n = 33 | **GG 4**  n = 18 | **GG 5**  n = 18 | **Total**  n = 149 |
| **Downgrading** | N/A | <5 | <5 | 7  (21%) | 8  (44%) | 5  (28%) | 20  (13%) |
| **Unchanged** | <5 | <5 | 32  (67%) | 17  (52%) | <5 | 13  (72%) | 67  (45%) |
| **Upgrading** | 14  (100%) | 17  (94%) | 16  (33%) | 9  (27%) | 6  (33%) | N/A | 62  (42%) |
| **2D** | | | | | | | |
| **sBx**  **RP** | **Non-malignant**  n = 208 | **GG 1**  n = 833 | **GG 2**  n = 1,837 | **GG 3**  n = 555 | **GG 4**  n = 267 | **GG 5**  n = 136 | **Total**  n = 3,836 |
| **Non-malignant** | N/A | <5 | 5 | <5 | 0 | 0 | 10  (0.26%) |
| **Downgrading** | N/A | N/A | 105  (5.7%) | 179  (32%) | 164  (61%) | 69  (51%) | 517  (16%) |
| **Unchanged** | <5 | 270  (32%) | 1,361 (74%) | 295  (53%) | 55  (21%) | 67  (49%) | 2,053 (54%) |
| **Upgrading** | 204  (98%) | 559  (67%) | 366  (20%) | 80  (14%) | 48  (18%) | N/A | 1,257 (33%) |


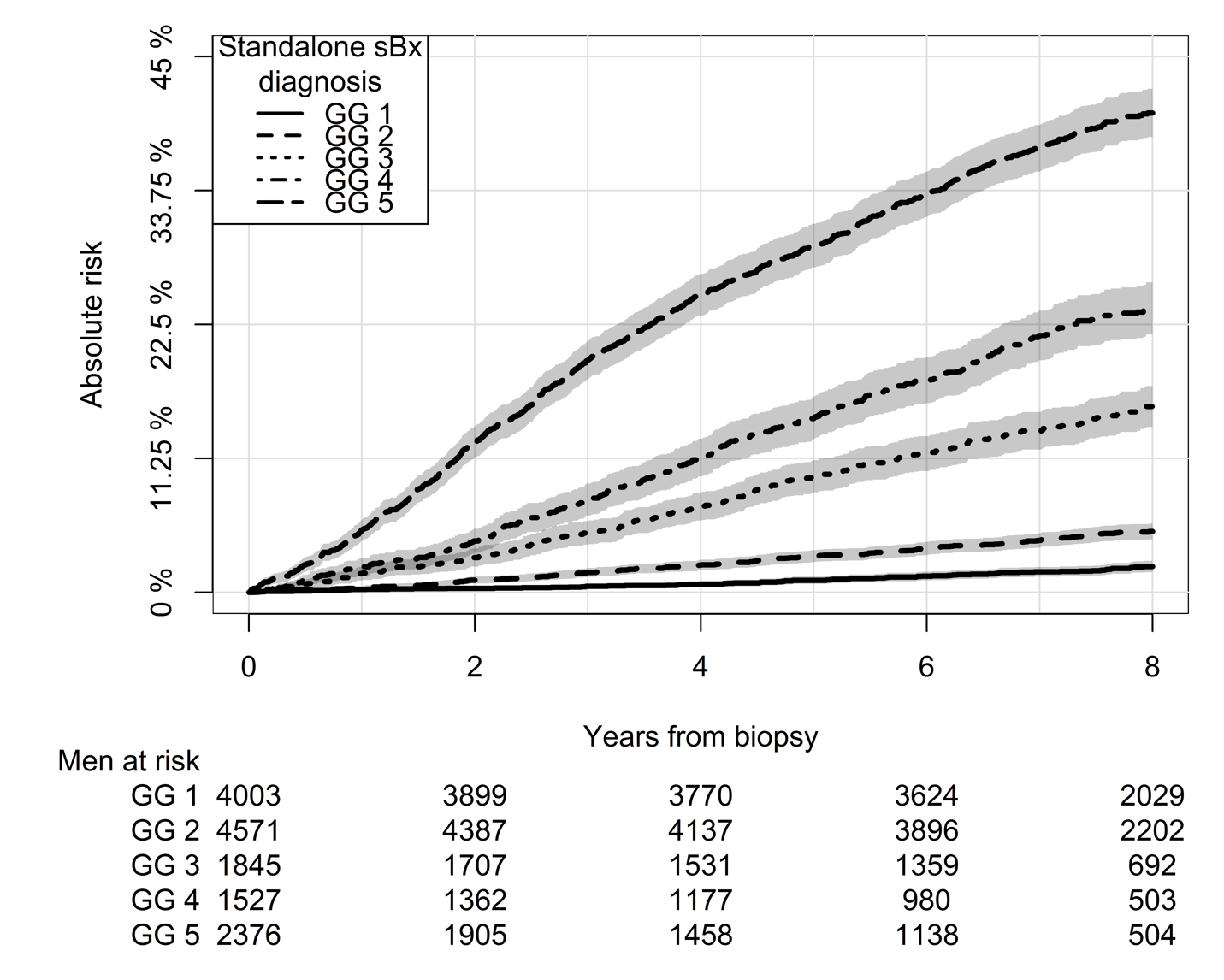


**Supplementary Figure 1:** Absolute risk of prostate cancer-specific death since the time of primary standalone systematic biopsy (sBx) stratified biopsy Gleason grading (GG).
